# Agar with embedded channels to study root growth

**DOI:** 10.1038/s41598-020-71076-w

**Published:** 2020-08-28

**Authors:** Azlan Abdul Aziz, Kai Boon Lim, Ena Kartina Abdul Rahman, Muhammad Hanafiah Nurmawati, Abu Samah Zuruzi

**Affiliations:** 1 Faculty of Engineering, Universiti Teknologi Brunei, Tungku Link, Gadong, BE1410 Brunei; 2 Singapore Accreditation Council, 2 Fusionopolis Way, Singapore, 138634 Singapore; 3 Department of Mechanical Engineering, College of Engineering, Alfaisal University, Riyadh, Kingdom of Saudi Arabia

**Keywords:** Soft materials, Techniques and instrumentation, Plant development, Mechanical engineering, Materials chemistry

## Abstract

Agar have long been used as a growth media for plants. Here, we made agar media with embedded fluidic channels to study the effect of exposure to nutrient solution on root growth and pull-out force. Black Eye bean (*Vigna Unguiculata*) and Mung bean (*Vigna Radiata*) were used in this study due to their rapid root development. Agar media were fabricated using casting process with removable cores to form channels which were subsequently filled with nutrient solution. Upon germination, beans were transplanted onto the agar media and allowed to grow. Pull-out force was determined at 96, 120 and 144 h after germination by applying a force on the hypocotyl above the gel surface. The effect of nutrients was investigated by comparing corresponding data obtained from control plants which have not been exposed to nutrient solution. Pull-out force of Black Eye bean plantlets grown in agar with nutrient solution in channels was greater than those grown in gel without nutrients and was 110% greater after 144 h of germination. Pull-out force of Mung bean plantlets grown in agar with and without nutrient solution was similar. Tap root lengths of Black Eye bean and Mung Bean plantlets grown in agar with nutrient solution are shorter than those grown without nutrient.

## Introduction

“Kanten”, as agar is known in Japanese, was first discovered from extracts of marine macroalgae, commonly known as seaweed, by Minoya Torazaemon in 1658^1^. Coastal communities in Asia including those in Malay Archipelago of South East Asia have long been extracting agar from genus Gracilaria as a food source. The name “agar” derives from the Malay lexicon to describe jelly(gel)-based food. “Agar” has more in common to “algae” from which the agar hydrogels are obtained. At the present time, production of agar from Gracilaria accounts for 14% of global seaweed aquaculture production with China, Indonesia, Malaysia, Philippines and Vietnam being major producers^2^.

Agar are polysaccharides with excellent hydrocolloidal properties suitable for many industrial applications^3^. Favorable attributes of agar include low production cost compared to alternative materials and ability to form reversible gels with acceptable mechanical properties at low concentration which is useful for applications in healthcare^4–6^. Furthermore, agar can be easily blended with other polymers to form composites with superior properties. Composite hydrogels of agar and gelatin have sufficient mechanical properties while blending with alginates results in smart hydrogels which respond to pH^7,8^.

Plant culture media contain nutrients, vitamins and other supplements needed for plant growth and development. Agar media is a suitable matrix for plant culture as agar are not digested by plant enzymes and do not react with supplements^9^. Plant culture media were developed from well-established bacteria growth media by adding nutrients needed to facilitate plant growth and development. Murashige and Skoog for example supplemented White’s modified agar medium with kinetin and indole acetic acid and observed yield of tobacco tissue culture increased 4–5 fold^10^. More recently, proliferation of nanomaterials in consumer and industrial applications has led to concern on the exposure of plant crops to nanomaterials and possible effects on plant development and food safety^11–13^. Researchers have used agar plant culture media dispersed with nanoparticles to investigate effect of exposure of plants to nanomaterials and interplay with local environment on plant development. Head lettuce plants grown in agar plant media containing ceria (CeO_2_) nanoparticles experienced phytotoxicity when concentration of ceria nanoparticles exceeds 500 mg L^−1^. At the roots of plants, reduction of CeO_2_ nanoparticles released Ce^3+^ ions which translocated to shoots. In contrast, phytotoxicity was reduced when phosphates were present in the agar plant media and is attributed to precipitation of Ce^3+^; a mechanism not observed when the plantlets were exposed to CeO_2_ nanoparticles in solution^14,15^. Agar media is also used for fundamental study of plant root development. In a recent study using agar media, root branching was observed to be significantly affected by availability of water around root structure. At root-agar regions where water potential is higher, biosynthesis results in accumulation and upward transport of auxin to maturation zones. This cascade of events promoted founder cell specification and eventually lateral root formation^16^. In addition, agar plate method has been used to study the effect of light on root growth using the improved agar-plate culture system (IPG)^17,18^. In the IPG method, roots are grown in shade or darkness; environment which mimics normal underground light conditions. Plants grown using IPG have significantly shorter total root length, lateral root length and root hair density, although their primary roots were longer compared to plant grown using traditional agar-plate culture medium.

In all prior work using agar plant media to investigate exposure, analytes such as nanoparticles and water were dispersed homogeneously in the agar. Agar is not sensitive to light and hence not amenable to patterning using photolithography. Furthermore, mechanical methods such as embossing are not suitable to pattern agar as it is non-rigid. A recent report by Josic et. al. fabricated channels by means of glass capillaries and wire as place holders in a gelatin/agar composite cross-linked with 1,1′carbonyldiimidazole. Diameters of channels embedded into the microfluidic device fabricated were 375 μm and 1,000 μm^19^. Although no application of the microfluidic device was demonstrated, optical clarity of the gel promises potential application as a see-through scaffold to study cell proliferation. Recent work has also focussed on integration of gel into lab-on-chip systems to leverage on the biocompatibility as well as amenability for molecules to diffuse through gel media, which promise new functionalities^20^.

To the best of our knowledge, this paper is the first to report use of fluidic channels in agar to study root architecture during plant development. Techniques that expose plants to analytes from predefined regions or in a fluid stream in the agar have not been developed. In this paper, we demonstrate the use of agar media with embedded channels to study plant root development and pull-out force after exposure to nutrients. Root architecture of beans have long been known to be affected by macronutrients such as phosphorus and nitrogen^21^. Moreover, root system of beans develops appreciably within a few days of germination making them suitable model plants to study. In this paper a novel approach is demonstrated using agar medium with embedded channels. The approach involves introducing a nutrient solution in the channels. Nutrients in the solution diffuse into the surrounding agar and induce root growth response^22^. The objective of this contribution is to demonstrate feasibility of agar with embedded channels to study root architecture development using nutrient solutions in the channels. Although nutrient solution is used here, the approach can be applied to study nanomaterials or any other analytes in fluid form or suspended in a fluid. Also, temporal and spatial variations of analytes can be studied by controlling the flow of nutrients in and through the design of channels.

## Results

### Integration of fluidic channels in agar media

**Figure 1:**
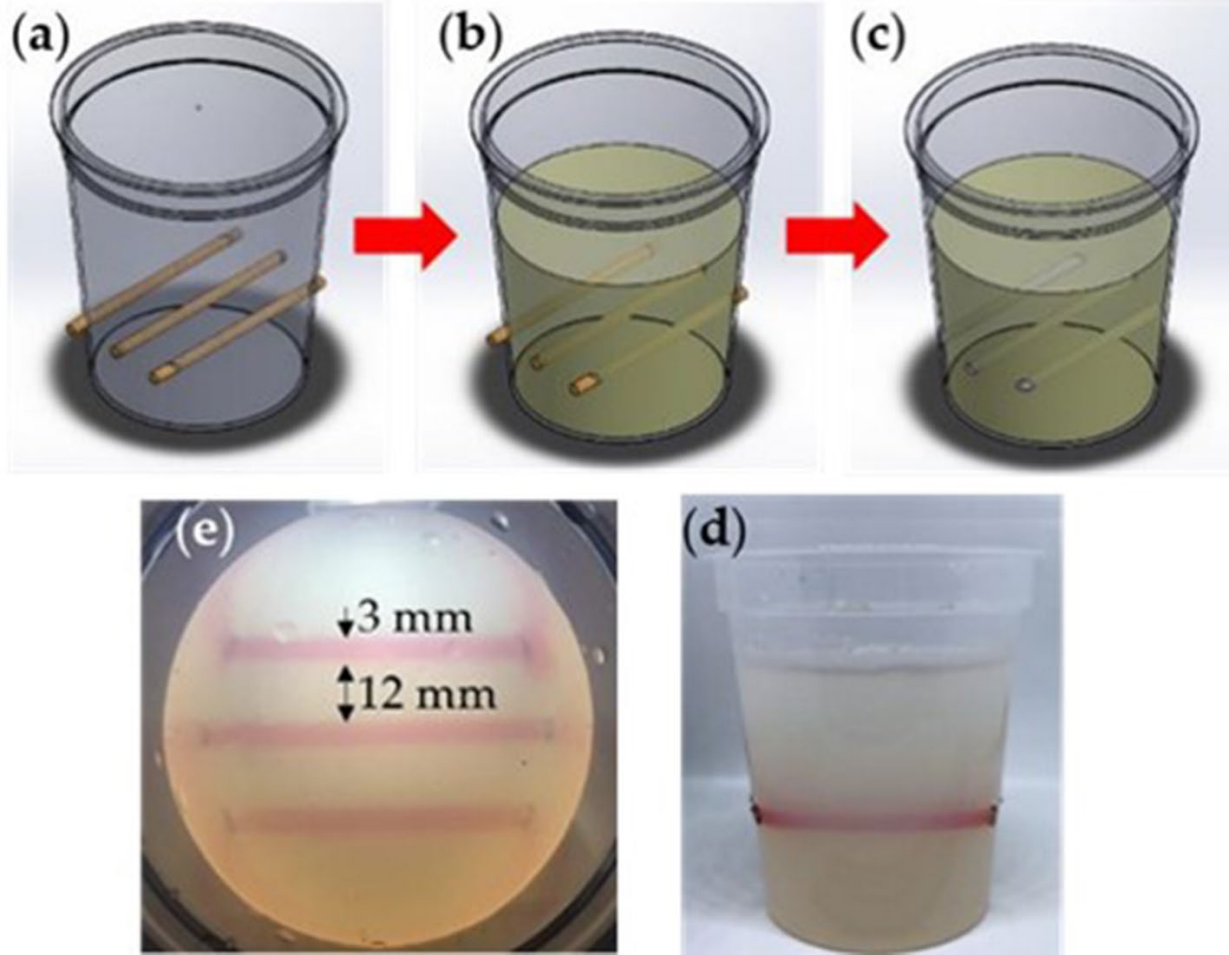
Fabrication of gel medium with embedded channels. (**a**) container with removable cores, (**b**) casting of gel and (**c**) removable of cores to form channels subsequently filled with aqueous nutrient solution. (**d**) and (**e**) are side and top views, respectively, of channels filled with aqueous red dye solution for contrast. (Image was created using Solidworks, Version 2017 SP, https://www.solidworks.com, and MS-Powerpoint, Version 16.16.22 (200,509) (Microsoft PowerPoint for Mac), https://www.microsoft.com).

Agar media with embedded fluidic channels were developed using a facile process as shown in Fig. 1a–c. The process involves pouring liquid agar solution (1 wt %) into polypropylene containers with dowels as place holders. Three dowels were preplaced at specific locations as place holders for channels and removed once the agar media have solidified. Figure 1d,e show channels that have been filled with aqueous-based red food dye to make them obvious. Water based red food dye and nutrient solutions do not leak from channels because of capillary forces and hydrophilicity of the agar. The volume of dye and nutrient solution in the channel decreased over time due to diffusion into the agar and evaporation from the ends of the channels; which can be refilled using a pipette.

**Figure 2:**
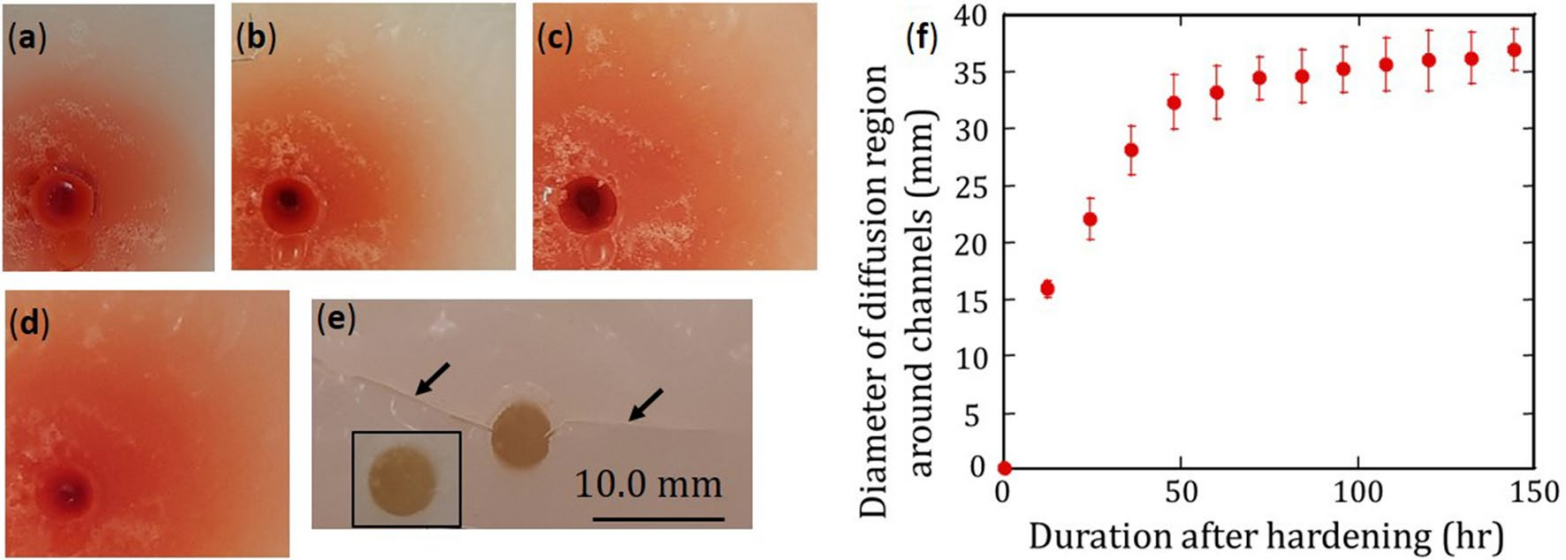
Region of agar around channels (at bottom left) showing diffusion of red die after nutrient solution has been introduced for (**a**) 12 h; (**b**) 24 h; (**c**) 48 h and (**d**) 72 h in the channels. (**e**) For agar with no solution in channels, cracks (arrows) propagate in agar from channel walls due to shrinkage after about 120 h; no cracks were observed at shorter time (inset). (**f**) Diameter of diffusion region around channels increases with time. (Image was created using MS-Powerpoint, Version 16.16.22 (200,509) (Microsoft PowerPoint for Mac), https://www.microsoft.com and using Kaleidagraph, Version 3.51, https://www.synergy.com).

Nutrients and water in the channels are expected to diffuse and permeate the agar medium. Figure 2 shows regions of agar around channels filled with nutrient solution added with red food dye to facilitate visualization. Regions of agar coloured by the red food dye increase in size with time after nutrient solution was introduced in channels. While it can be expected that rates of diffusion for red dye and nutrients in agar are different, Fig. 2 clearly shows embedded channels can be used to contain nutrient solution and will not collapse or blocked due to swelling of agar. In addition, agar with nutrient solution-filled channels do not degrade by cracking for the range of time investigate in this study, although more studies at much longer times are needed. In contrast, for agar specimens whose channels were not filled with nutrient solution, cracks were observed after about 120 h after hardening.

### Mechanical properties of gels

**Figure 3:**
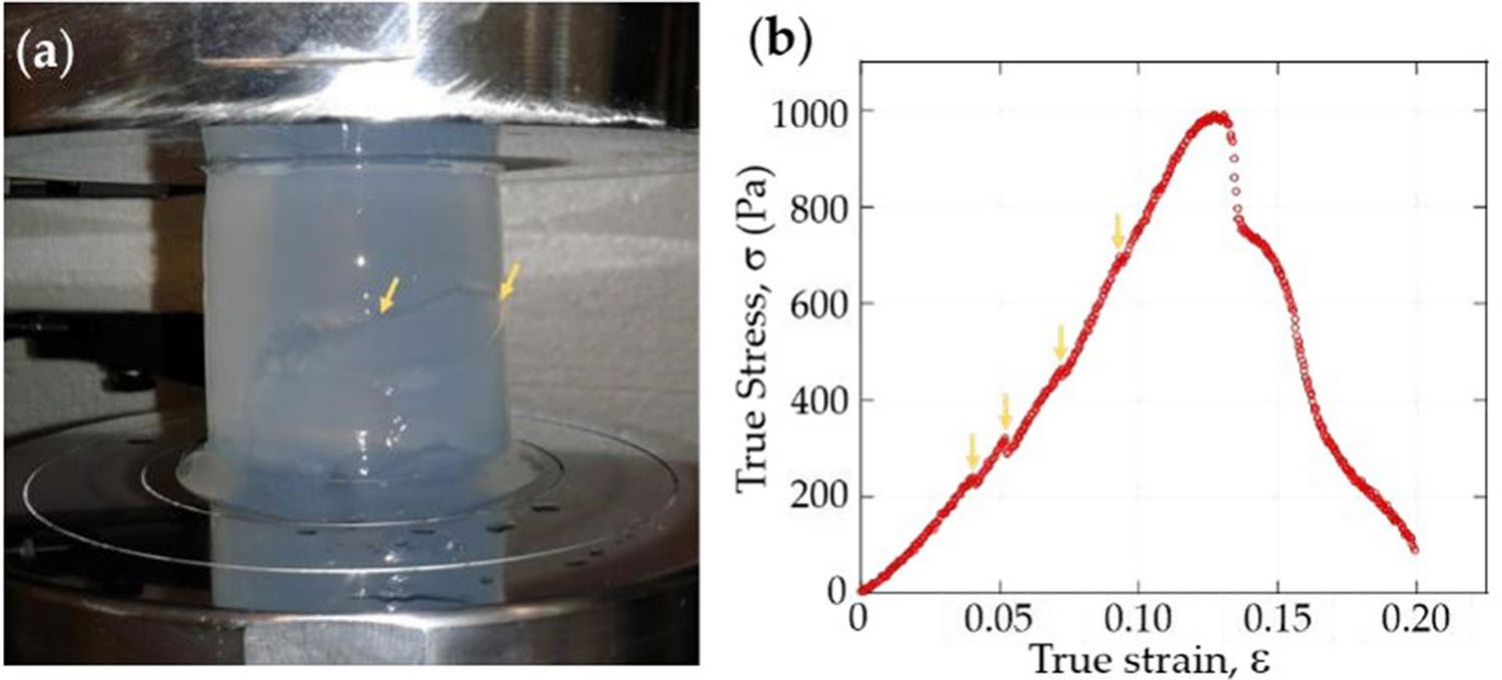
Characterization of mechanical properties of agar. (**a**) Agar specimens were compressed between steel platens until failure (orange arrows) to give (**b**) plots of true stress versus true strain. (Image was created using Kaleidagraph, Version 3.51, https://www.synergy.com and MS-Powerpoint, Version 16.16.22 (200,509) (Microsoft PowerPoint for Mac), https://www.microsoft.com).

Uniaxial compression tests were performed using an Instron 5,565 universal testing machine equipped with a 5 kN load cell. Compression tests were performed by placing agar specimens between steel platens. Agar specimens tested were of the same age as those used in the growth of bean plants; that is after 96, 120 and 144 h after germination. Cylindrical specimens 7.5 cm high and 5.3 cm in diameter were placed between platens and compression was carried out at a loading rate of 1 mm/min until the agar specimens experienced gross failure as shown in Fig. 3a. Load (force) and displacement data were transformed to true stress versus true strain plots as shown in Fig. 3b.

A typical true stress versus true strain curve during compression of an agar specimen begins with a monotonic increase of true stress until about 0.04 strain. At ε > 0.04, a few samples exhibit flaws on the surface such as tearing. Development of such flaws results in a drop in true stress before increasing again. Other flaws (highlighted by orange arrows) that develop thereafter will also result in corresponding drops in true stress which recovers, increases and ultimately, reaches a maximum value. The maximum true stress value corresponds to the point where, in most cases, significant tearing which propagates (fracture) in the gel was observed. This maximum true stress value recorded is denoted as the true stress at fracture and the corresponding strain as the true strain at fracture. Beyond the true strain at fracture, a much lower applied true stress results in significant tearing of the agar. True stress increases slightly more rapidly than a linear relation with true strain, which suggest occurrence of a mild strain hardening mechanism.

### Growth of bean plants and tap root length

Root architecture of bean plants has been widely shown to be affected by nutrient availability^23^. In this study, bean plants were exposed to a commercially available plant nutrient solution. The nutrient solution has a pH of 6.1 and the composition is shown in Table 1.

**Table 1:** Composition of nutrient solution. (Table was created using MS-Word, Version: 16.16.22 (200509) (Microsoft Word for Mac), https://www.microsoft.com).

Component	Concentration (g/l)
Potassium (K_2_O)	40
Nitrogen	20
Sulphate (SO_4_^2−^)	20
Humic acid	10
Phosphorus (P_2_O_5_)	6
Magnesium (MgO)	5
Iron (Fe) chelated	0.8

**Figure 4:**
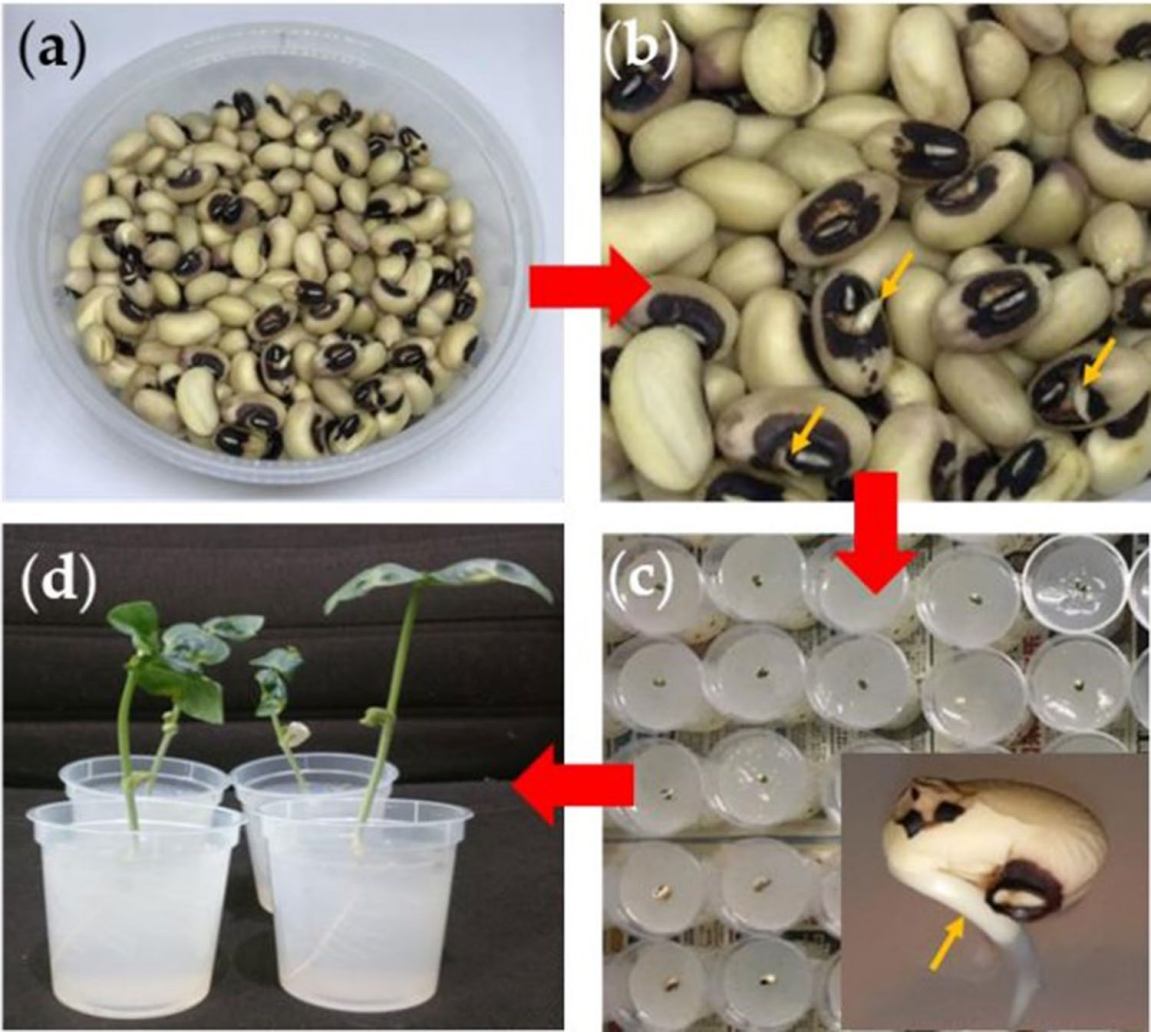
Growth of bean plants. Starts from (**a**) germination after soaking for a few hours; (**b**) until radicles (orange arrows) are observed; (**c**) followed by transplanting on the surface of the agar; inset shows with radicles touching and subsequently growing into the gel. (**d**) Black eye bean plants at 144 h after germination. (Image was created using MS-Powerpoint, Version 16.16.22 (200,509) (Microsoft PowerPoint for Mac), https://www.microsoft.com).

Bean plants were investigated in this study as they have been used in many prior studies on how root development in plants response to changes in nutrients availability^21,23^^.^^24^. Black Eye bean (Vigna Unguiculata) and Mung bean (Vigna Radiata) plants were grown in agar media cast into polypropylene containers. To study the effect of exposure to nutrients on each type of beans, two sets of plants were grown. One set of plants were grown in agar media in which the channels were filled with nutrients while other set were not exposed to any nutrients. Figure 4a,b shows germination and radicle formation of beans, respectively. Root caps are placed in contact with the agar surface during transplanting as shown in Fig. 4c. Germinating beans develop shoots after growing under sunlight for a few days as shown in Fig. 4d.

**Figure 5:**
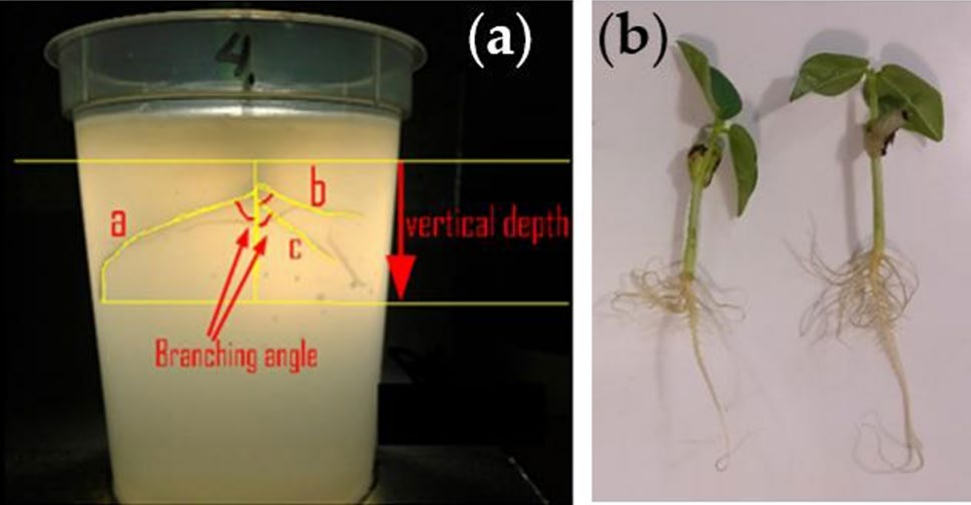
Measurement of tap root length. Performed (**a**) in-situ by digitally analyzing images and (**b**) ex-situ on excised plants after pull-out tests. (Image was created using MS-Powerpoint, Version 16.16.22 (200,509) (Microsoft PowerPoint for Mac), https://www.microsoft.com).

Root system of beans develop from a radicle. The root tip pushes into the gel as cells in the region of meristematic activity divide, elongate and enlarge with a concomitant extension and widening of the radicle as the root cap grows into the agar to form the primary or tap root. Lateral roots are formed from branches emanating from the tap root. In this study, phenotype of interest is the tap root length. Figure 5a,b shows measurement of root length parameters in-situ using a freely available software and after pull-out test.

### Pull-out force of plants after various times of germination

**Figure 6:**
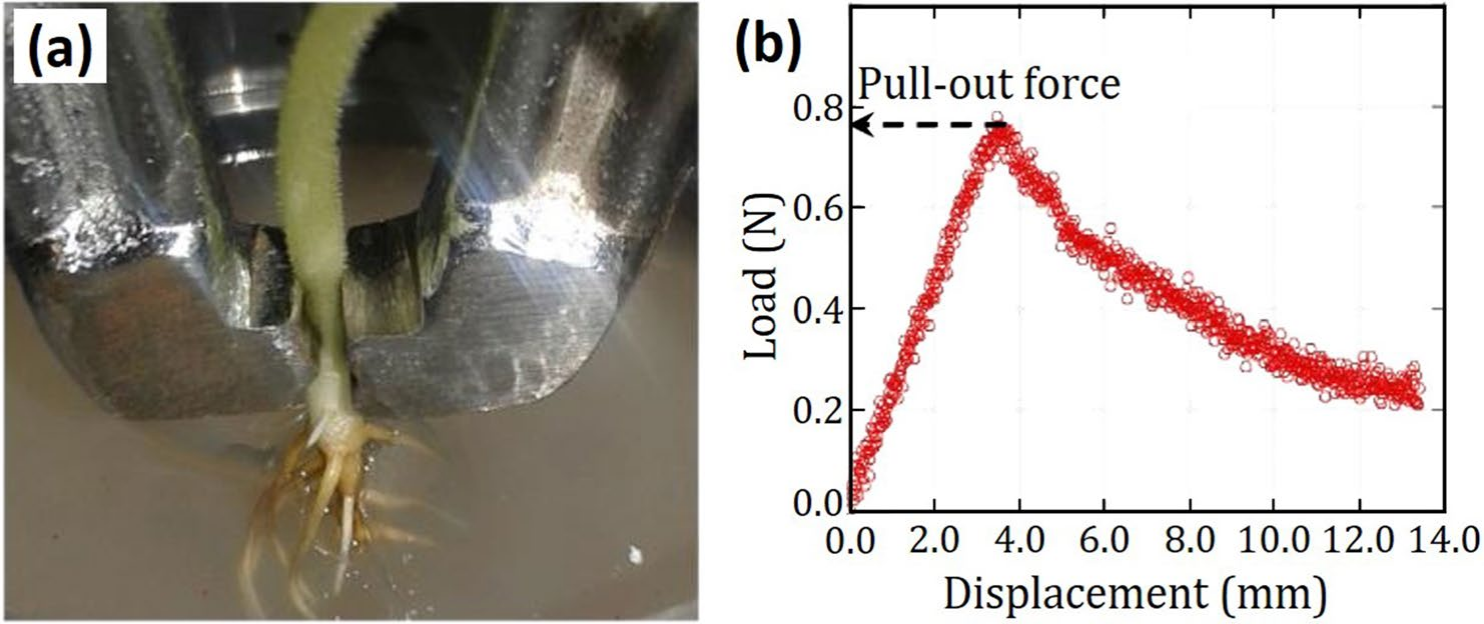
Determination of pull-out force were carried out at 96, 120 and 144 h after germination. (**a**) Clamping the portion of hypocotyl just above the gel surface to give load versus displacement plots; (**b**) The pull-out force is defined as the maximum load recorded during the pull-out test. (Image was created using Kaleidagraph, Version 3.51, https://www.synergy.com and MS-Powerpoint, Version 16.16.22 (200,509) (Microsoft PowerPoint for Mac), https://www.microsoft.com).

Pull-out of bean plants were performed using the same Instron 5,565 universal testing machine with clamping fixtures rather than platens. Tests were done by applying an axial tensile force at the hypocotyl just above the gel surface. This region of the plant has the largest width and able to withstand a gripping force from a mini-clamp without deforming, as shown in Fig. 6a. As an axial tensile load is applied, the stiff hypocotyl transmits the load to the root system and the agar which experience a displacement. A typical load–displacement curve has two distinct segments as shown in Fig. 6b. The first portion is characterized by a linear increase in load with displacement until a maximum load is reached. The maximum load is defined as the pull-out force and further displacement was achieved at a lower load. In most cases, the pull-out force was observed to be the point at which the root system, as a whole, debonds from and were pulled out of the agar media.

The tap root length and pull-out force of Black Eye beans and Mung beans under exposure to nutrients or otherwise are shown in Table 2. The average values and standard deviations of measurements from 10 plants were calculated for bean type and condition. Tap root length of Black Eye beans and Mung bean plants are affected by presence of nutrients in channels. Tap root length of plants are longer when not exposed to nutrients. However, only the pull-out force of Black Eye beans seems to be affected by the presence of nutrients; the pull-out forces are greater when there are nutrients. In contrast the average pull-out force for Mung beans exposed to nutrients or not, are similar.

**Table 2:** Effect of nutrient exposure on bean plant traits (*n* = 10 for bean plant and condition).

Treatment	Time after Germination (h)	Black Eye bean		Mung bean	
		With nutrient	Without nutrient	With nutrient	Without nutrient
Tap root length (cm)	96	4.49 ± 1.31	9.39 ± 1.20	1.47 ± 0.50	4.87 ± 1.89
	120	5.74 ± 2.10	11.13 ± 1.28	2.17 ± 0.97	4.87 ± 1.82
	144	5.93 ± 2.22	11.59 ± 1.65	2.45 ± 1.09	7.15 ± 3.80
Pull-out force (N)	96	0.83 ± 0.21	0.50 ± 0.17	0.52 ± 0.26	0.64 ± 0.14
	120	1.29 ± 0.74	0.70 ± 0.14	0.70 ± 0.35	0.79 ± 0.18
	144	2.08 ± 0.93	0.75 ± 0.19	0.91 ± 0.25	0.87 ± 0.09

## Discussion

Agar are hydrophilic polysaccharides and have significant water content^25^. Agar will lose its water content through evaporation when exposed to ambient resulting in shrinkage. Mechanical stress is generated in agar during shrinkage which causes morphological deformation such as surface wrinkling and crack formation^26^. Water loss affects local micro-environment in the hydrocolloid which results in changes in the mechanical properties of agar. When used as a platform to study plants, agar with embedded channels will be exposed to ambient condition which will invariably result in mass loss due to evaporation of water from the agar. Embedded channels in agar need to be robust against gross structural deformation including rupture or blockage of channels. Hence it is imperative that mass loss of agar as well as dimensions and integrity of channels are studied.

**Figure 7:**
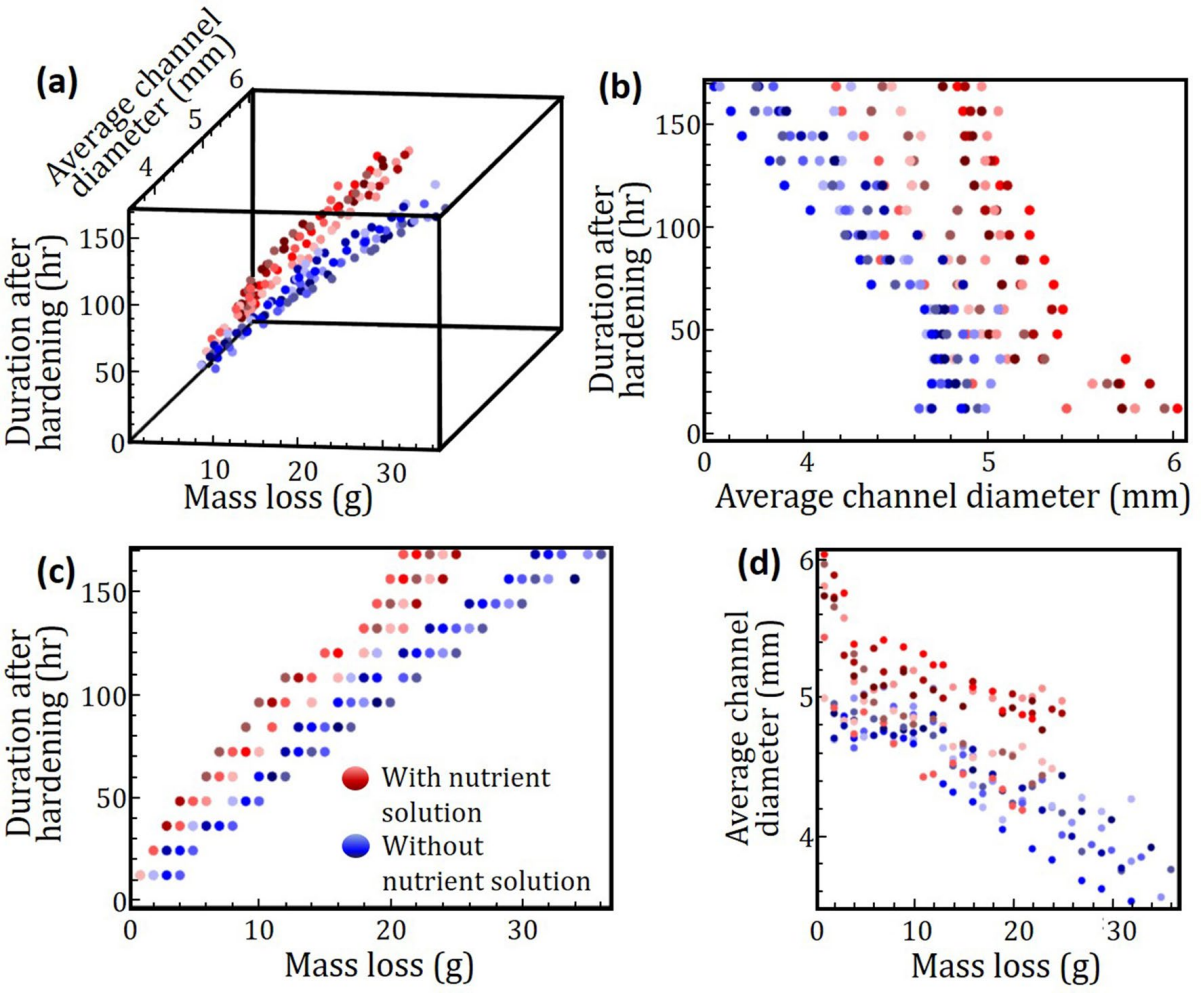
Agar specimens containing nutrient solution are represented by spheres of reddish hue, while those that do not are represented by spheres of  blueish hue. Seven hues of red (or blue) denote seven agar specimens containing (or not containing) nutrient solution, respectively. (**a**) Three-dimensional representation of average channel diameter and mass loss at various duration after hardening. (**b**) In general, average diameter of channel with nutrient solution is larger than those without nutrient solution and, at short durations, even greater than core diameter used to form channels. (**c**) Mass loss for all agar specimens increases with duration after hardening with agar containing nutrient solutions showing a lower mass loss. (**d**) Average channel diameter decreases with mass loss due to shrinkage. (Image created using Mathematica, Version: Home Edition, https://www.mathematica.com and MS-Powerpoint, Version 16.16.22 (200,509) (Microsoft PowerPoint for Mac), https://www.microsoft.com).

Mass loss of agar and average channel diameter varies with duration after the agar hardens as shown in Fig. 7. Mass loss of agar increase with duration after hardening. Furthermore, mass loss of agar without nutrient solution (represented by blueish spheres) are higher than those with nutrient solution. When placed in ambient, water evaporation causes agar to lose mass. For agar with nutrient solution in channels (represented by reddish spheres), mass loss is compensated by diffusion of nutrients and water resulting in a lower mass loss, Fig. 7a,c. Lower mass loss means shrinkage is reduced and mechanical stress due to shrinkage is reduced as well. Consequently, agar with nutrients in channels do not exhibit crack formation for the range of duration studied. In contrast, cracks were observed in agar without nutrients in channels after 120 h. Maximum mass loss recorded for agar with and without nutrients was 27% and 36%, respectively.

In general, average channel diameter in agar decreases with duration after hardening as shown in Fig. 7b. Also, average channel diameter for agar without nutrients is lower than those for agar with nutrients. Higher shrinkage experienced by agar without nutrients means greater shrinkage stress acting on the channel which causes the diameter to decrease. A similar trend was observed for channels with nutrients although the rate at which average channel diameter decreases is slower. Furthermore, at shorter durations the average channel diameter is greater than the diameter of dowels (5.0 ± 0.1 mm) used to form the channels. This is attributed to swelling of agar at channel walls followed by erosion (or dissolution) into nutrient solution. As agar loses water through evaporation, reduction in channel diameter due to shrinkage negates and overwhelms the swelling effects of agar in the channel; hence the lower rate at which average channel diameter decreases.

Pull-out force of plants from soils has been widely used to study the effect of plant roots on soil stability^27,28^. Pull-out force of plants depends on many factors including root architectural features such as tap root length and branching points as well as root mechanics such as tensile strength and elastic modulus. Mechanical properties of the medium in which a plant is growing to some extent affect the pull-out force through interfacial friction between roots and medium^29^. Root architecture such as helical growth of root is affected by hardness of agar^30^. Hence, mechanical properties of agar such as tensile strength, strain at break and elastic modulus can be expected to affect pull-out force if there is significant variation during the duration of the study. Furthermore, agar degrades when exposed for prolonged duration to environment^31^. Hence, mechanical properties of agar specimens in the present study were investigated.

**Figure 8:**
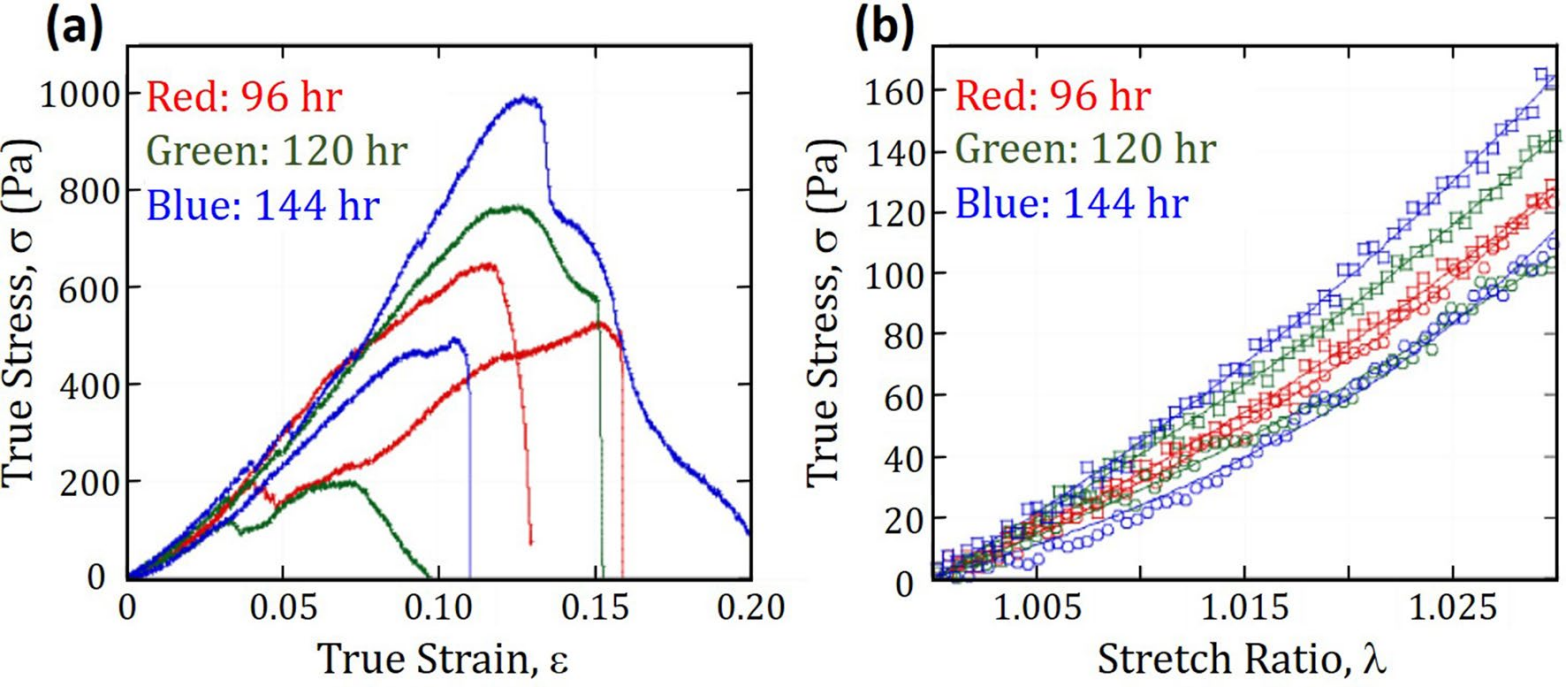
Variation of true stress. (**a**) True stress versus true strain curves of agar specimens during compression test; (**b**) True stress versus stretch ratio (1 < *λ* < 1.03 corresponds to 0 < ε < 0.03). (Image created using Kaleidagraph, Version 3.51, https://www.synergy.com).

Figure 8a shows true stress versus true strain curves of agar, done in duplicates (labelled as specimens A and B) after 96, 120, and 144 h after hardening, identical to duration for pull test of plants. There is wide variation in the true stress at fracture between samples and there is clearly no trend with respect to time after hardening. However, for 0 < ε < 0.04, which is close to the strain at which pull-out occurs, true stress curves for all specimens are very close and increases monotonically in a non-linear manner; except when tears happen on the surface, which reduce true stress slightly. The similarity suggests there is minimal effect from the agar media on the trends observed for pull-out force and tap root length.

The Ogden relation is often used to model non-linear mechanical behaviour of materials exhibiting elastic property with strain hardening when undergoing large strains^32–34^. The relation is expressed in Eq. (1):

1 $$\sigma =\frac{2\mu }{\alpha }\left({\lambda}^{\alpha }-{\lambda}^{-\frac{\alpha }{2}}\right)$$

where  $\sigma=$ True stress,  $\mu=$ Shear modulus of gel,  $\alpha=$ Strain hardening parameter,  $\lambda=$ Stretch ratio defined as Euler’s number ($e$) raised to the power of true strain ($\varepsilon$), = ${e}^{\varepsilon }.$

Figure 8b shows a plot of the true stress versus stretch factor of agar specimens during compression for 1 < $\lambda$  < 1.03 which corresponds to 0 < ε < 0.03. The shear modulus and strain hardening parameter of the get can be extracted by fitting experimental curves to Eq. (1) and are shown in Table 3. It was found that 673 Pa < μ < 1,358 Pa while 28 < α < 56 and data indicate no noticeable difference between mechanical properties of agar after various times of hardening. The shear moduli extracted using the Ogden formalism are close to those reported in the literature; being only about one order of magnitude smaller^35^. This could be due to differences in the method of measurement and molecular weight of agarose in agar used in the present and past study^36^. Strain hardening parameters are large and positive which suggest agar behaves, to a large extent, linear elastically and confirms minimal strain hardening occurred in the agar during compression.

**Table 3:** Parameters extracted from compression tests of agar.

Time after germination (h)	Specimen	True stress at fracture (Pa)	True strain at fracture (%)	Shear modulus, μ (Pa)	Strain hardening parameter, α
96	A	526	15.1	927	40
96	B	648	11.4	1,047	32
120	A	200	7.18	882	30
120	B	767	12.6	1,246	28
144	A	495	10.5	673	56
144	B	992	13.1	1,358	31

**Figure 9:**
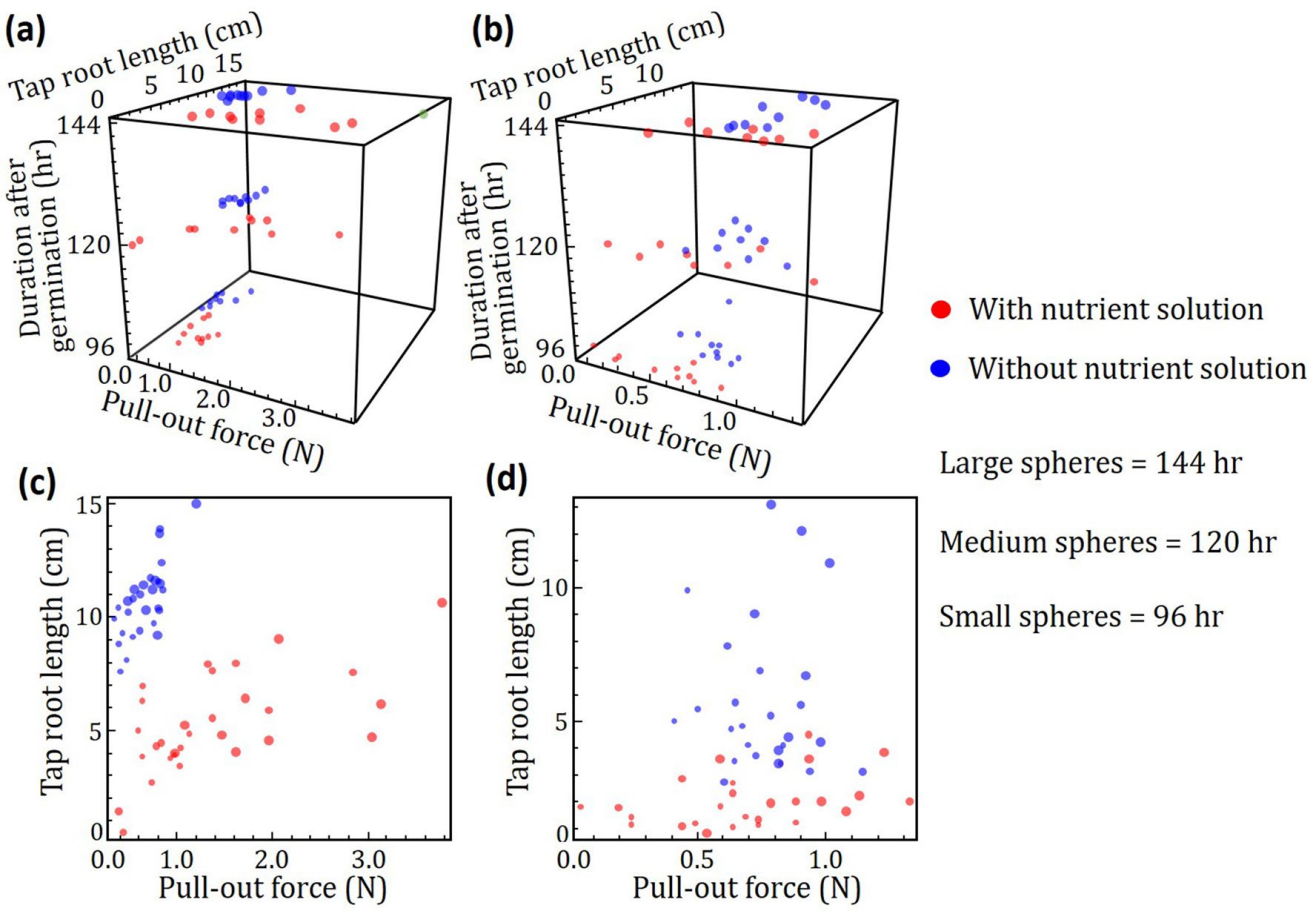
Three-dimensional representation of pull-out force and tap root length at various times after germination for (**a, c**) Black Eye beans and (**b, d**) Mung beans. Plants grown with and without nutrients are represented by red and blue spheres, respectively. Size of spheres increases with time after germination. Black eye bean and Mung bean plants grown with no nutrients in the fluid channels have longer tap root length. There is no statistical difference in the pull-out forces of Mung bean plants grown with and without nutrients. In contrast, Black Eye bean plants grown with nutrients in channels have significantly larger pull-out force than those grown without and the difference increases with time after germination. (Image created using Mathematica, Version: Home Edition, https://www.mathematica.com and MS-Powerpoint, Version 16.16.22 (200,509) (Microsoft PowerPoint for Mac), https://www.microsoft.com).

Figure 9a-d shows three-dimensional distributions of tap root length and pull-out force of all plants tested. Distributions of Black Eye bean plants not exposed to nutrients (blueish spheres) are clustered towards smaller pull-out force and larger tap root length as shown in Fig. 9a,c. There is no overlap between the reddish (with nutrient condition) and blueish (without nutrient condition) spheres indicating there is clear effect of nutrients on the tap root length and pull-out force of Black Eye bean plants. Furthermore, it is clear that distributions of tap root length and average pull-out force shift to larger values with time after germination. For Mung beans as shown in Fig. 9b,d, plants exposed to nutrients (reddish spheres) are distributed at smaller tap root lengths. However, the distributions of Mung bean plants exposed and not exposed to nutrients are similar for the pull-out force axis.

**Figure 10:**
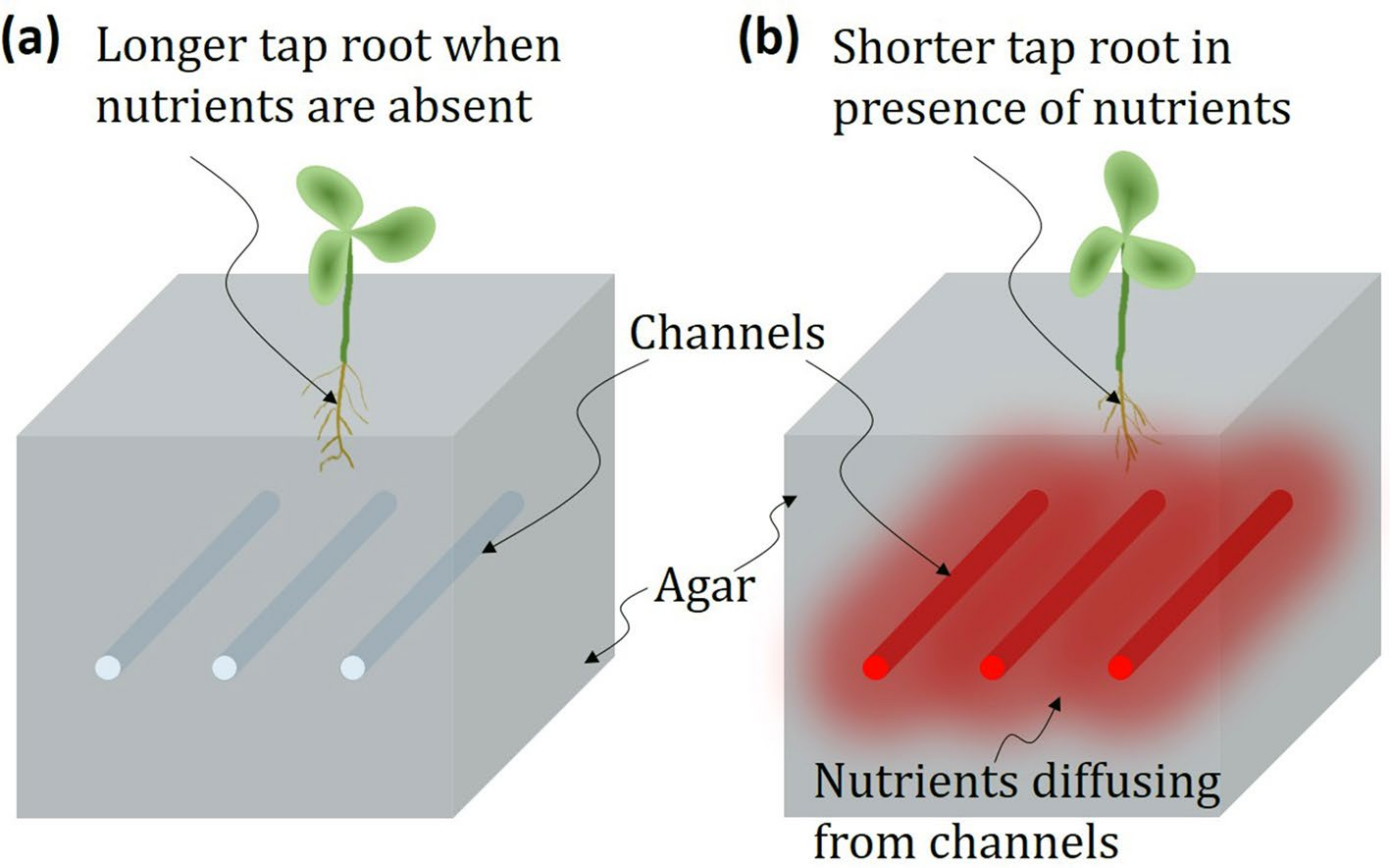
Schematic of agar (**a**) without and (**b**) with nutrient solution in embedded channels. Diffusion of nutrients in agar results in shorter tap root. This response localizes root development to regions where nutrients are readily available. In contrast, tap root length of plants in agar media without nutrients are longer. (Image created using MS-Powerpoint, Version 16.16.22 (200,509) (Microsoft PowerPoint for Mac), https://www.microsoft.com).

Nutrient depletion (or availability) has been known to affect plants^37,38^. Soils rich in phosphorus have been known to be induce root proliferation^39^. Plant nutritional studies using agar benefits from agar’s amenability to diffusion as well as its ability to protect nutrients from sorption and precipitation^22,40^. Results of the present study clearly show agar with embedded channels can be used to study root development in bean plants. Nutrients diffuse radially from embedded channels into and permeates surrounding agar as shown in Fig. 10. Consequently, nutrients are more readily available nearer the surface for plants in agar with nutrient solutions. Root plasticity of Mung bean and Black Eye bean plants exposed to nutrients from channels responds by developing shorter tap roots which localize root development to regions where nutrients are readily available. In contrast, those in agar without nutrient in channels develop longer tap root length. Images and measurements of the two root structures are shown in Figs. 5b and 9, respectively. Nutrient deficiency has been reported to produce faster-growing taproots and diminished root branching among a few species of dicots, of which beans belong^41^. Results of this study are in agreement with many prior reports on the response of plants to variations in the nutrient supply in a wide range of species^42–44^.

Plants optimize allocation of resources by directing root growth within the region of soil that yields the most benefit in terms of nutrient capture. Shorter tap roots improve efficiency, since nutrients are readily available nearer to the surface and obviate the need for longer tap roots which incur a larger C expenditure (root growth). Water, a resource associated at deeper soil depths and hence requires longer tap roots, is readily available in agar media. Hence, bean plants in this study are displaying phene prioritization for nutrients, over water, acquisition. For Black Eye bean plants, although the nutrients in the channels induce shorter tap roots, the pull-out forces for these plants are in general larger. This suggests other phenes, besides tap root length, have a more dominant effect on pull-out force. Nevertheless, other factors such as bonding at the root/agar interface, lateral roots and tortuosity of roots also affect pull-out force and future studies will systematically study their effects.

## Conclusions

A procedure was developed to construct agar with embedded fluid channels. Agar media were then used to study the effect of nutrients on root architecture development of germinating Black Eye beans and Mung beans. Root development of both type of bean plants is affected by nutrients that diffused into the agar from nutrient solution in channels. Tap root length of bean plants exposed to nutrients in the channels are shorter than those not exposed. The pull-out force of Black Eye beans plants exposed to nutrients in the channels are larger than those not exposed. However, no noticeable difference between pull-out force was observed for Mung beans exposed and not exposed to nutrients. Agar loses water due to evaporation which results in mass loss and shrinkage; shrinkage brings about a reduction in average channel diameter. A smaller reduction in channel diameter and mass loss were observed for channels filled with nutrient solution. Mechanical properties of agar do not degrade up to a week after casting.

## Methods

### Agar media preparation

Agar powder was purchased from a commercial supplier (Swallow Globe, Singapore) and used without further modification. Agar hydrogels (1 wt%) were prepared by adding water at 80 °C to agar powder. The mixture was stirred continuously until all agar powder were dissolved and a homogeneous solution formed. Three types of agar were prepared. Agar specimens for compression tests were prepared by pouring agar solution into polypropylene cylinders of 5.3 cm diameter. Solidified gel was then removed from containers and cut such that it is 7.5 cm high. Agar media for compression tests were stored at the same conditions as agar media used for plant growth.

Agar media for plant growth were prepared by pouring agar solution into polypropylene containers with three 3.0 ± 0.3 mm diameter dowel pins placed across each cup, spaced 12 mm apart. All dowel pins were placed 30 ± 1 mm above the base of the container. The axis of the middle dowel intersects the long axis of the container and the other two dowels are placed 1.5 ± 0.1 cm away from the middle dowel. Each container was filled with up to 7.0 ± 0.2 cm height of agar solution with the mass of the agar being 100.0 ± 0.5 g. Once the agar has hardened, dowel pins are removed leaving channels in the solid agar. To study the effect of exposure to nutrients on each type of beans, two sets of plants were grown. One set of plants were grown in agar media in which the channels were filled with nutrients while other set were not exposed to any nutrients. Accordingly, nutrient solution was immediately dispensed into channels using a pipette for the first set while for the other set, their channels were left unfilled. Nutrient solution did not leak out from the channels due to capillary forces and hydrophilic nature of the agar surface. Nutrients in the channels are expected to diffuse into and permeates the agar medium and refilled using pippette.

Agar specimens were also prepared to study mass loss, channel diameter and cracking due to evaporation. For this type of agar media, agar solution was poured into polypropylene containers with one 5.0 ± 0.1 mm diameter dowel pin. Nutrient solution used in the present study is colourless and transparent making it difficult to visualize diffusion in the agar. Red food dyes were added to the nutrient solution. Mass and images of agar regions around a channel for agar specimens with unfilled and filled with nutrient solution were captured every 12 h. ImageJ software was used to calculate the average diameter of channels from 8 diameter measurements^45^. ImageJ was also used to determine the average diameter of the diffused region around channels from measurements of 7 specimens for each condition.

### Characterization of plant roots

Black Eye bean (Vigna Unguiculata) and Mung bean (Vigna Radiata) were used as their root system develop rapidly and can be studied within the time frame of the project. Bean plants were grown from seeds. Germination was initiated by soaking seeds in water for 12 h. Seeds were then removed and placed on sterile napkins at room ambient for 10 h after which seeds with radicles were placed on an agar media such that the root cap is touching the surface of agar. Agar is prepared fresh and used right after hardening and channels filled with nutrient solution. Each container of agar contains one bean. The bean plant is then allowed to grow in a chamber while exposed to nutrients in the channel. Agar media were then placed in sterile growth room and exposed to natural sunlight through a window between 7.00 am to 6.00 pm and under simulated sunlight from lamps at other times of the day.

### Determination of pull-out force

An Instron 5,565 universal testing machine equipped with a 5 kN load cell and running the Bluehill software for data collection, visualization and experiment control was used to determine pull-out force. Plants were tested at 96, 120 and 144 h after germination at 25℃ at relative humidity between 55 to 60%. To determine pull-out force of a plant, its hypocotyl just above the gel surface was gripped with interlocking and corrugated mini-clamp attached to the crosshead. The container of agar media was held firmly in place by a rigid Perspex test frame. Pull-out tests were carried out at a cross-head velocity of 1 mm per min and terminated manually when the root of plants has been fully dislodged from agar specimens. The load and strain measurement accuracies for the Instron 5,565 system are rated at ± 0.5% of readings, for both types of measurements^46^.

### Determination of mechanical properties of agar

Mechanical properties of agar media such as true stress and true strain at fracture were determined using compression tests. Agar media were tested in duplicates after 96, 120 and 144 h after casting, similar to duration for pull-out test of plants. Relative humidity and temperature at test conditions were identical to those during pull-out force determination. Axial compression tests were performed using the same universal testing machine used to determine the pull-out test. To perform compression tests, steel platens were attached to the crosshead instead of mini-clamp. Compression tests were carried out at a cross-head velocity of 1 mm per min and terminated manually when agar specimens exhibited gross failure.
